# The Impact of Left Ventricular Performance and Afterload on the Evaluation of Aortic Valve Stenosis: A 1D Mathematical Modeling Approach

**DOI:** 10.3390/bioengineering10040425

**Published:** 2023-03-28

**Authors:** Cemre Çelikbudak Orhon, Nikolaos Stergiopulos, Stéphane Noble, Georgios Giannakopoulos, Hajo Müller, Dionysios Adamopoulos

**Affiliations:** 1 Laboratory of Hemodynamics and Cardiovascular Technology, Institute of Bioengineering, Ecole Polytechnique Fédérale de Lausanne, 1015 Lausanne, Switzerland; 2 Faculty of Medicine, University of Geneva, 1211 Geneva, Switzerland; 3 Department of Internal Medicine, Division of Cardiology, Hopitaux Universitaires de Genève (HUG), 1205 Geneva, Switzerland

**Keywords:** aortic valve stenosis, transaortic valvular pressure gradient, left ventricular systolic and diastolic function, total vascular resistance, total arterial compliance

## Abstract

The transaortic valvular pressure gradient (TPG) plays a central role in decision-making for patients suffering from severe aortic stenosis. However, the flow-dependence nature of the TPG makes the diagnosis of aortic stenosis challenging since the markers of cardiac performance and afterload present high physiological interdependence and thus, isolated effects cannot be measured directly in vivo. We used a validated 1D mathematical model of the cardiovascular system, coupled with a model of aortic stenosis, to assess and quantify the independent effect of the main left ventricular performance parameters (end-systolic (E_es_) and end-diastolic (E_ed_) elastance) and principal afterload indices (total vascular resistance (TVR) and total arterial compliance (TAC)) on the TPG for different levels of aortic stenosis. In patients with critical aortic stenosis (aortic valve area (AVA) ≤ 0.6 cm^2^), a 10% increase of E_ed_ from the baseline value was associated with the most important effect on the TPG (−5.6 ± 0.5 mmHg, *p* < 0.001), followed by a similar increase of E_es_ (3.4 ± 0.1 mmHg, *p* < 0.001), in TAC (1.3 ±0.2 mmHg, *p* < 0.001) and TVR (−0.7 ± 0.04 mmHg, *p* < 0.001). The interdependence of the TPG left ventricular performance and afterload indices become stronger with increased aortic stenosis severity. Disregarding their effects may lead to an underestimation of stenosis severity and a potential delay in therapeutic intervention. Therefore, a comprehensive evaluation of left ventricular function and afterload should be performed, especially in cases of diagnostic challenge, since it may offer the pathophysiological mechanism that explains the mismatch between aortic severity and the TPG.

## 1. Introduction

Calcific aortic valve disease (CAVD) is one of the prevalent cardiovascular diseases in western countries [1,2,3]. Over the past three decades, there has been a substantial increase in CAVD-related incidence and mortality cases, and the total number of CAVD-related deaths more than doubled from 1990 to 2019 [1]. Age is an important risk factor for aortic stenosis, and its prevalence in western countries is approximately 25% in people above 65 years of age, while it rises to 50% in those above 85 years of age [2,4,5]. Since late treatment significantly increases the mortality rate, timely and accurate diagnosis today becomes even more important as populations become older [6,7].

The transaortic valvular pressure gradient ([TPG], (the difference in pressure between the left ventricle and the ascending aorta), has been traditionally used as a hemodynamic marker of valvular stenosis and, as such, it is a part of the echocardiographic examination in clinical routine [8]. When the mean TPG exceeds 40 mmHg, aortic stenosis is classified as severe [9]. Valve replacement is recommended for severe aortic stenosis cases with a class I indication when patients are symptomatic or when in the absence of clear symptoms and where systolic left ventricular ejection is <50% without another cause, or when patients experience demonstrable symptoms on exercise testing [7].

Any effort to physiologically contextualize the TPG in a specific hemodynamic environment requires a deep understanding and precise quantification of the independent effects of each potential determinant on the flow and pressure gradients. This exploration, however, is challenging since the markers of cardiac performance and afterload present a high physiological interdependence, and thus, the isolated effects cannot be measured directly in vivo. Secondly, no pharmacological intervention is able to specifically affect one variable without interfering with any other, and thus, independent effects cannot be experimentally quantified.

Mathematical models are great tools for simulating complex and interconnected physiological conditions in vivo. A 1D modeling of the cardiovascular system provides wave propagation analyses with a low computational cost [10]. Up to the present, 1D analyses have been extensively used to simulate the hemodynamical characteristics of the cardiovascular system, and the simulated pressure and flow waveforms are successfully validated with the in vivo measurements [10,11,12,13]. It has been shown that these models can be used not only to study complex hemodynamic mechanisms but also to develop integrative diagnostic tools and methods by offering a very flexible platform of in silico experimentation [14,15,16]. Since the magnitude of the TPG is normally insignificant, these models generally assume that the aortic valve is ideal, which means it does not impose any pressure gradient.

One of the pioneering studies on the simulation of aortic stenosis was performed by Clark [17]. In this study, the TPG was modeled as the sum of the local inertial, convective inertial and frictional forces caused by the blood flow as it passes through the stenosis [17]. The results were comparable to those from animal experiments. However, the aortic valve area was assumed to be constant through the ejection phase in the model, and this was simulated with the partial occlusion of the proximal aorta of animals [17]. The relationship between the TPG and flow has been constructed extensively by using the generalized Bernoulli equation, and similar to the Clark [17], many studies have considered the aortic valve area to be fixed, assuming that the opening and closure of a valve is fast during the ejection phase [18,19,20]. While the rapid opening of the aortic valve is a valid assumption in healthy individuals, this is not the case in those suffering from stenosis due to the prolonged opening time [21]. Studies have shown that the ratio of acceleration time—the required time to reach a maximum aortic flow—to the total ejection time increases as the severity of aortic stenosis increases [21,22,23]. Virag et al. [24] used the lumped parameter model of the left ventricle and arterial system coupling, and the motion of the aortic valve area was defined as a function of volume displaced by leaflets during the opening and closure phases in systole. Later, Aboelkassem et al. [25] extended the work of Virag et al. [24], adding the effect of sinus vortices that are created downstream to the aortic valve due to diverging geometrical characteristics. However, the assessment of the displaced volume by the leaflets is not easily accessible in vivo, and it makes this method challenging to apply. Recently, Laubscher et al. [26] proposed a more advanced dynamic valve model by including the effect of Reynold’s number on the TPG and then compared it with the previous model of Korakianitis et al. [27]. Both models solved the differential equations of valve dynamics, and the opening of a valve was modeled as a function of the angular position of the leaflets [26,27,28]. These models require various geometrical valve parameters, such as the angular position and thickness of the leaflets, and this is difficult to be obtained with conventional imaging techniques. Mynard et al. [29] used the Bernoulli equation to relate the TPG and flow, and it characterized the motion of a valve as a function of the instantaneous TPG and the state of a valve [30]. In another study of Mynard et al. [30], this aortic valve model, without imposing an aortic stenosis condition, has been coupled with a 1D cardiovascular system model, and the results were able to capture the characteristics of aortic pressure and flow waveforms in vivo [30].

In fact, the TPG is not just a simple index determined by the characteristics of the aortic valve; rather, it is a more complex parameter that depends on the flow as well. However, despite its broad use in clinical practice, very few studies have explored the interaction between the aortic TPG and the hemodynamic environment in which the measurement is performed [31,32,33]. It is known that compliance and elastance are important indexes responsible for the main physiological changes in biological tissues, and they are also used to interpret the progression of a disease or the outcomes of the treatments [34,35,36]. As a flow-dependent measure, physiological determinants of the cardiac output (systolic and diastolic ventricular function, arterial compliance, vascular resistance, and wave reflections) are expected to have an important impact on the aortic TPG, and thus, they should be taken seriously under consideration when interpreting measurements [34,37,38,39]. Furthermore, aortic stenosis generally coexists with arterial stiffening, which further increases the left ventricular afterload [31,38], and they are both the consequences of similar pathophysiological progress [34]. Clinical and experimental data show that, particularly in low flow states (either because of decreased myocardial contractility or increased afterload), the TPG may be low despite the presence of severe aortic stenosis, leading to diagnostic inaccuracies and therapeutic delays [40,41,42,43,44,45]. Repeating the measurement after pharmacological modulation of the cardiac output (dobutamine or nitrates infusion) is a classic strategy to address this issue by transiently increasing the stroke volume (SV); however, many exams still remain inconclusive, while significant side effects may be observed due to the administration of the vasoactive drugs [8,46,47]

To our knowledge, the independent effects of the left ventricular parameters and afterload indices on the TPG have, so far, never been studied. To better understand the interplay between myocardial performance, afterload and aortic stenosis, we have coupled the mathematical aortic valve stenosis model with a 1D mathematical model of the cardiovascular system and validated the results. In this study, for the first time, the independent effect of the left ventricular, systolic and diastolic function (end-systolic elastance (E_es_) and end-diastolic elastance (E_ed_), accordingly), total vascular resistance (TVR) and total arterial compliance (TAC) on the TPG for different levels of aortic valve stenosis is aimed to be quantified.

## 2. Materials and Methods

### 2.1. 1D Mathematical Model of the Cardiovascular System

In the present study, a 1D mathematical model of the cardiovascular system was implemented. The technical characteristics and clinical validation of the model have been presented in detail previously [48]. In summary, the model is based on the 1D form of continuity and longitudinal momentum of the Navier–Strokes equations and a constitutive law describing the relation between cross-sectional and distending pressure. The model includes 103 segments of the main systemic arteries. The compliance of the arterial segments is modeled as a function of location and pressure, as proposed by Langewouters et al. [49]. Based on the approach of Holenstein et al. [50], the local arterial lumen area depends on the viscoelastic and non-linear characteristics of the artery wall. Wall shear stress is calculated according to the Witzig-Womersley theory [51].

### 2.2. Boundary Conditions

**Figure 1:**
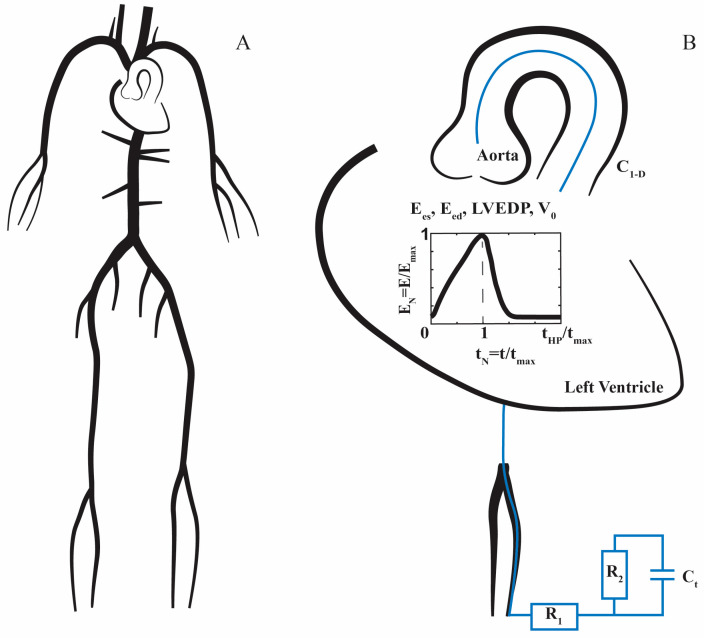
(**A**) 1D model of the cardiovascular system with main systemic arteries; (**B**) at the proximal site, the left ventricular is modeled according to the varying elastance model. The instantaneous elastance of the left ventricle is calculated for a given E_es_, E_ed_, heart period (t_HP_) and maximum elastance time (t_max_) by exploiting the normalized varying elastance (E_N_). Left ventricular end-diastolic pressure (LVEDP) and dead volume (V_0_) are the additional parameters needed to construct the pressure–volume loop of a patient. At the distal boundaries, the 1D model is coupled to a three-element Windkessel model, which includes terminal compliance (C_t_), proximal resistance (R_1_) and distal resistance (R_2_). Compliances of the main systemic arteries are represented as C_1-D_.

At the distal sites, peripheral arteries are coupled with a three-element Windkessel model, which includes terminal compliance (C_t_), proximal resistance (R_1_) and distal resistance (R_2_), as depicted in Figure 1, while the proximal aorta is coupled with the left ventricle, which is modeled according to the varying elastance model, as described by Sagawa et al. [52]. According to this approach, the instantaneous elastance of left ventricle E(t) is defined with the following relation:

(1) $$E(t)=\frac{P_{\mathrm{LV}}\left(t\right)}{V_{\mathrm{LV}}(t)\;-V_{d}}$$

where $P_{\mathrm{LV}}(t)$ is the instantaneous left ventricle pressure, $V_{\mathrm{LV}}(t)$ is the left ventricle’s instantaneous volume and $V_{d}$ is the dead volume. By using the normalized varying elastance curve provided by Senzaki et al. [53], E(t)  is calculated for a given E_es_, E_ed_, heart period, and maximum elastance time of a given case/patient, as described in Figure 1B [54]. The entire set-up of the equations is implicitly solved for the entire cardiac cycle, providing the pressure and flow waveforms throughout the entire arterial tree.

### 2.3. Stenotic Aortic Valve Model

The aforementioned 1D cardiovascular model assumes an ideal aortic valve with no TPG through the aortic valve. In order to simulate aortic stenosis, the aortic valve model proposed by Mynard et al. [29] was used after modifications according to the Young et al. model [55,56]. According to Mynard et al. [29], by neglecting viscous losses, the instantaneous pressure gradient across the aortic valve is given as the sum of inertial losses and turbulence losses associated with the divergent distal part of the stenosis:

(2) $$\Delta P(t)=L\cdot \frac{\mathrm{dQ}(t)}{\mathrm{dt}}+\beta \;\cdot \;Q(t)\;\cdot \;\left|Q(t)\right|$$

where Q(t) is the instantaneous transaortic valvular flow and L is the blood inertance term with having an effective valve length of $l_{\mathrm{eff}}$, blood density ρ and effective area $A_{\mathrm{eff}}$:

(3) $$L=\frac{\rho \;\cdot \;l_{\mathrm{eff}}}{A_{\mathrm{eff}}}$$

$A_{\mathrm{eff}}$ defines the instantaneous valve area. In our study, we define $A_{\mathrm{eff}}$ as:

(4) $$A_{\mathrm{eff}\;}={\;A}_{\mathrm{mes}}\;\cdot \;\zeta \left(t\right)$$

where ζ(t) defines the state of the valve with 0 ≤ ζ(t) ≤ 1, ζ=0 and ζ=1, representing a completely closed and open valve, respectively. $A_{\mathrm{mes}}$ is the measured area of the aortic valve or the maximum area when the valve is completely open.

In order to estimate the pressure loss due to the turbulence in the divergent part more completely, the Bernoulli term β in the Mynard et al. [29] model is changed according to Young et al. [55,56].

(5) $$\beta =\frac{K_{t}\;\cdot \;\rho }{2}{\left(\frac{1}{A_{\mathrm{eff}}}-\frac{1}{A_{o}}\right)}^{2}$$

where $K_{t}$ is an empirical coefficient and chosen as 1.5, as proposed by Young et al. [55,56]. $A_{o}$ represents the unobstructed area of the channel. Since pressure losses occur at the diverging side due to flow separation and turbulence, the unobstructed area of the channel for the aortic valve can be approximated as a cross-sectional area of the ascending aorta, where flow is diverged after passing the aortic valve. Therefore, the size of the ascending aorta also affects the pressure loss through the aortic valve: the larger the ascending aorta, the larger the turbulence-related pressure losses.

The rates of valve opening and closure are given by the following equations as proposed by Mynard et al. [29], respectively:

(6) $$\frac{d\zeta \left(t\right)}{\mathrm{dt}}=\left[1-\;\zeta \left(t\right)\right]\;\cdot \;K_{\mathrm{vo}}\;\cdot \;\Delta P(t)$$

(7) $$\frac{d\zeta \left(t\right)}{\mathrm{dt}}=\zeta \left(t\right)\;\cdot \;K_{\mathrm{vc}}\;\cdot \;\Delta P$$

where $K_{\mathrm{vo}}$ and $K_{\mathrm{vc}}$ are the opening and closure coefficients, which are assumed to be equal in our simulations. By starting with the lower values, the opening/closure coefficient value of the valve is iteratively increased until it can reach the fully open state for a given ejection time of a patient.

### 2.4. Validation of the Coupled Model

**Figure 2:**
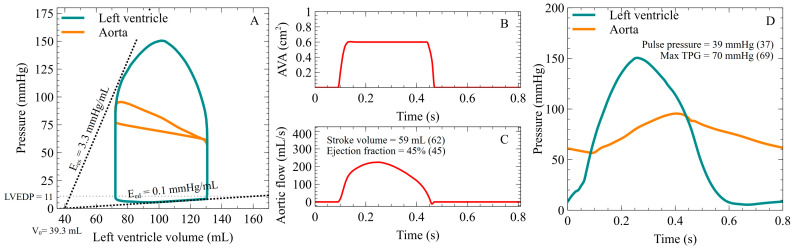
1D mathematical model coupled with aortic valve stenosis model for the prediction of TPG. (**A**) Predicted left ventricular and aortic pressure–volume curve from the model (input variables E_es_ = 3.3 mmHg/mL, E_ed_ = 0.1 mmHg/mL, V_0_ = 39.3 mL and LVEDP = 11 mmHg, and maximal AVA = 0.6 cm^2^). (**B**) Aortic valve opening as a function of time derived by the aortic valve stenosis model. (**C**) Aortic flow, generated by the coupled 1D mathematical and the aortic valve stenosis model. (**D**) Performance of the model in predicting TPG as compared to the actual measured values from the literature [57].

We tested the performance of the coupled 1D cardiovascular system/aortic stenosis model against the in vivo data provided by the study of Dekker et al. [57]. In this work, the left ventricular pressure–volume loops (PV) of patients with severe aortic stenosis were acquired by inserting a conductance catheter with a pressure sensor in the left ventricular cavity during transient inferior vena cava occlusion. Aortic pressure was also measured simultaneously. Data for a specific patient (E_es_, E_ed_, left ventricular end-diastolic pressure (LVEDP), dead volume (V_0_) and AVA) were acquired and given as an input to our model (Figure 2 panels A and B) in order to create a patient-specific simulation. In our cardiovascular model, the TAC of a patient is the total sum of the compliances of the main systemic arteries (C_1-D_) and the terminal compliance (C_t_), while the TVR is the sum of the proximal resistance (R_1_) and the distal resistance (R_2_), as described in Figure 1B. The patient’s TVR and TAC were first estimated by using an initial TVR value, estimated by the ratio of the mean aortic pressure and divided by the cardiac output and TAC value as the ratio of SV to pulse pressure. In the following step, values are iteratively increased or decreased within the physiological limits until the predicted mean aortic flow is comparable to the one measured. The simulation results of the model are presented by comparing them with the measured values in parentheses in Figure 2 (panel C for the aortic flow waveform and panel D for the left ventricle and aortic pressure waveforms). The estimations of the model were validated by comparing the results with the measured values of pulse pressure and the maximum TPG and are presented in Figure 2D.

### 2.5. Simulation Strategy of the Aortic Stenosis Cases

The objective of the present study was to evaluate and quantify the independent effect of the left ventricular performance and afterload indexes on the TPG for a given aortic stenosis. A total of 10 cases of critical aortic stenosis (AVA = 0.6 cm^2^) were created, presenting a progressive increase of E_es_ for a range of physiological values (min 0.5 mmHg/mL to max 6 mmHg/mL) [48,58]. Since we aimed to estimate the independent effect of E_es_ on the TPG, the diastolic performance of the left ventricle (E_ed_) and afterload (TVR and TAC) remained constant during these simulations. In order to explore the potential interaction with the severity of the aortic stenosis, the same hemodynamic settings were applied for 30 additional cases but with different values of AVA (1.0 cm^2^, 1.5 cm^2^ and 2.0 cm^2^, *n* = 10 cases per AVA stenosis level). The same strategy was applied for an incremental change in E_ed_ (min 0.03 mmHg/mL to max 0.31 mmHg/mL) [48,59], a change in TVR (min 0.6 mmHg×s/mL to max 1.8 mmHg×s/mL) [60] and change in TAC (min 0.5 mL/mmHg to max 2 mL/mmHg) [60]. Simulations were run by letting each key parameter vary from the lowest to the highest possible, with 10 evenly spaced values in the predefined range, while at the same time, maintaining all other parameters as constant. In addition, since the TAC and TVR do not change independently from each other in vivo [61], we simulated 40 additional cases with a progressive increase in afterload but with the TAC and TVR being coupled according to a hyperbolic relation linking the two variables.

It is important to highlight that since a change in any parameter may affect others across the entire arterial system (e.g., an increase of E_es_ enhances the pressure in the arterial system, which in turn decreases the TAC), during our simulations, we iteratively increased or decreased the parameters of the arterial tree until they converged to the initial set values within a given error threshold. Finally, it should be noted that the capacity of the model to simulate situations with an extreme discrepancy between the TAC and TVR while keeping the left ventricular performance parameters unaffected is limited since the ventricular–arterial interaction would counterbalance these effects by modifying E_es_ in the physiological cases.

### 2.6. Echocardiography

An expert cardiologist performed a complete transthoracic echocardiography on the patient at rest according to established guidelines [62]. Transesophageal echocardiography was performed under the dobutamine administration one week later. The offline evaluation was carried out at the related workstation (IntelliSpace Cardiovascular 5.1; Philips Medical Systems Nederland B.V., Best, The Netherlands). The velocity waveform in the left ventricle outflow tract was obtained by pulsed-wave Doppler, while the aortic velocity waveform was acquired by aligning the continuous wave doppler with the aortic valve. The aortic valve area and pressure gradient were calculated by using the continuity equation and the simplified Bernoulli equation, respectively [62].

### 2.7. Statistical Analysis

**Figure 3:**
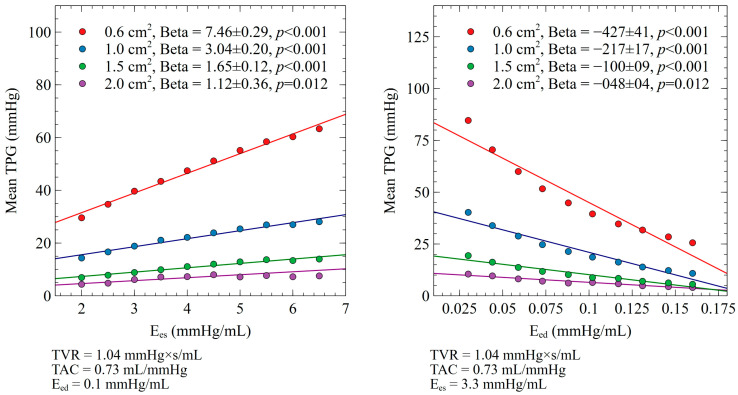
Impact of E_es_ and E_ed_ on mean TPG for different levels of aortic valve stenosis.

**Figure 4:**
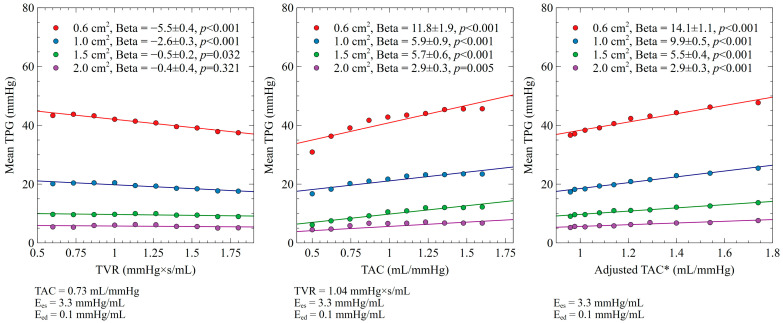
Impact of TVR and TAC on mean TPG for different levels of aortic stenosis. Adjusted TAC*: coupled TAC according to its hyperbolic relation with TVR [61].

The associations between the individual determinants and mean TPG were assessed separately for each level of maximum AVA (0.6 cm^2^, 1.0 cm^2^, 1.5 cm^2^ and 2.0 cm^2^) by the use of linear regression analysis. The values are expressed as either the regression beta coefficient ± standard error (Figure 3 and Figure 4) or the pressure gradient change for every 10% increase of the independent variable (Table 1). Statistical significance was assumed at a two-sided P-value level of 0.05. Statistical analysis was performed in IBM SPSS statistics (IBM Corp. Released 2020. IBM SPSS Statistics for Windows, Version 27.0. Armonk, NY, USA: IBM Corp.).

## 3. Results

### 3.1. E_es_ and E_ed_ Impact on Mean TPG

The impact of the E_es_ and E_ed_ changes on the mean TPG for different levels of aortic stenosis is presented in Figure 3 and Table 1. A decrease in left ventricular myocardial contractility, assessed by the E_es_, was associated with a lower mean TPG (AVA 0.6 cm^2^ (beta 7.46 ± 0.29, *p* < 0.001), AVA 1.0 cm^2^ (beta 3.04 ± 0.20, *p* < 0.001), AVA 1.5 cm^2^ (beta 1.6 ± 0.12, *p* < 0.001), and AVA 2.0 cm^2^ (beta 1.12 ± 0.36, *p* < 0.012)). A significant interaction with the AVA was seen, with the relation between E_es_ and the mean TPG being stronger in the most severe aortic stenosis cases. Accordingly, an increase in left ventricular stiffness/relaxation, assessed by the E_ed_, was associated with a lower mean TPG for a given AVA (AVA 0.6 cm^2^ (beta −427 ± 41, *p* < 0.001), AVA 1.0 cm^2^ (beta −217 ± 17, *p* < 0.001), AVA 1.5 cm^2^ (beta −100 ± 9, *p* < 0.001), and AVA 2.0 cm^2^ (beta −48 ± 4, *p* < 0.012)). A significant interaction with the AVA was seen, with the relation between E_ed_ and the mean TPG becoming stronger in parallel with the progression of aortic valve stenosis severity.

### 3.2. TVR and TAC Impact on Mean TPG

The impact of the TAC and TVR changes on the mean TPG for different levels of aortic stenosis are presented in Figure 4 and Table 1. An increase in TVR was associated with a decrease in the mean TPG for a given AVA (AVA 0.6 cm^2^ (beta −5.5 ± 0.35, *p* < 0.001), AVA 1.0 cm^2^ (beta −2.6 ± 0.33, *p* < 0.001), and AVA 1.5 cm^2^ (beta −0.5 ± 0.23, *p* = 0.032)). This was not seen in cases with an AVA of 2.0 cm^2^ (beta −0.4 ± 0.35, *p* = 0.321). A significant interaction with the aortic valve area was seen, with the relation between TVR and the mean TPG becoming stronger as the aortic stenosis grew more severe. Accordingly, an increase in TAC was associated with an increase in the TPG for a given AVA (AVA 0.6 cm^2^ (beta 11.8 ± 1.9, *p* < 0.001), AVA 1.0 cm^2^ (beta 5.9 ± 0.9, *p* < 0.001), AVA 1.5 cm^2^ (beta 5.7 ± 0.6, *p* < 0.001), and AVA 1.0 cm^2^ (beta 2.9 ± 0.3, *p* = 0.005)). A significant interaction with the AVA was seen, with the relation between TAC and the pressure gradients becoming stronger with the progression of aortic severity. Similar results were obtained when coupled TAC and TVR values were used as input variables (Figure 4 and Table 1).

### 3.3. Relative Contribution of E_es_, E_ed_, TVR, and TAC on Mean TPG

When expressed as a 10% change from the baseline, in patients with an AVA of 0.6 cm^2^, the E_ed_ change was associated with the most important effect on the TPG (−5.6 ± 0.5 mmHg, *p* < 0.001), followed by E_es_ (3.4 ± 0.1 mmHg, *p* < 0.001), TAC (1.3 ± 0.2 mmHg, *p* < 0.001), and TVR (−0.7 ± 0.04 mmHg, *p* < 0.001). Similar classifications were noted with a higher AVA; however, the magnitudes of the effect seem to become weaker as aortic stenosis severity decreases.

### 3.4. SV and Mean TPG Relationship for a Given AVA

**Figure 5:**
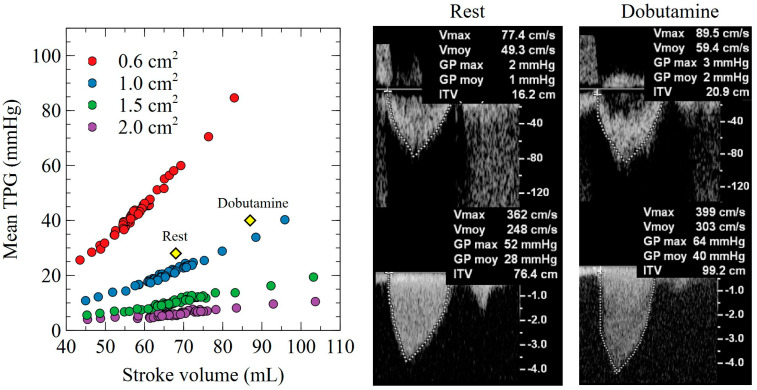
Association between SV and mean TPG for different levels of AVA. Echocardiographic evaluation of a patient with severe aortic stenosis at rest and after dobutamine infusion (12.5 ug/min/kg). The myocardial recruitment observed after dobutamine leads to an increase in stroke volume (from 67 mL to 87 mL) with a concomitant increase in mean TPG (from 28 mmHg to 40 mmHg). The AVA remained stable, suggesting a true severe aortic valve stenosis (AVA 0.88 cm^2^ for an LVOT diameter measured at 23 mm). Model predictions were accurate for both rest and dobutamine hemodynamic conditions.

Since E_es_, E_ed_, TVR, and TAC independently affect the TPG through a flow-dependent manner, we explored the association directly between SV and the mean TPG (Figure 5). A significant increase in the mean TPG was seen with an increase in SV for any given AVA. A significant interaction with an AVA severity is also observed, with the dependence being stronger as the aortic valve severity increases.

## 4. Discussion

In the present study, a 1D mathematical model of the cardiovascular system, coupled with an aortic valve stenosis model, was used in order to assess and quantify, for the first time, the independent effect of the main left ventricular performance variables, as well as afterload indices, on the TPG in the presence of aortic stenosis. The main conclusions of the present study could be summarized in the following points: (1) Left ventricular diastolic function (E_ed_) is, at least, as important as left ventricular myocardial contractility (E_es_) in determining the TPG in the presence of aortic stenosis; (2) TAC, as well as TVR, affect the TPG independently for a given aortic stenosis level; and (3) the interaction between the left ventricular performance, afterload indices, and the TPG becomes stronger as aortic valve severity increases.

The relationship between low left ventricular contractility and a low TPG in patients with severe aortic stenosis is well known. In the case of a low ejection fraction (e.g., post-myocardial infarction), an underestimation of the TPG is expected due to a low flow state (classical low-flow, low-gradient aortic stenosis) [40]. As shown in Figure 5, our model was capable of accurately predicting the relationship between SV and the mean TPG, assessed by transthoracic echocardiography for a patient with severe aortic stenosis. The administration of dobutamine, in this particular case, increased SV (myocardial recruitment) and concomitantly increased the mean TPG to a level comparable to our model predictions. (Figure 5). It is important to note that two configurations, the before and after dobutamine administrations, lie on the same hypostatical curve that shares the same valve area. In the case of pseudo-stenosis, since the increased stroke volume will be able to open the aortic valve further, it would be expected that the case after dobutamine will shift to another curve, having a larger valve area.

In addition to left ventricular contractility, our results highlight the major importance of diastolic function for the evaluation of the TPG since the independent effect of the E_ed_ was even higher than the one of E_es_, especially in patients with critical AVA. This is in accordance with clinical studies showing strong associations between the diastolic dysfunction indices and the presence of severe aortic stenosis without an increased TPG and with a normal ejection fraction (paradoxical low-flow, low-gradient aortic stenosis) [63]. This is of major clinical relevance since aortic stenosis and diastolic dysfunction often coexist and evolve parallel with aging [64,65]. It follows that a comprehensive evaluation of diastolic dysfunction should be part of the routine examination when evaluating aortic valve disease since it may significantly blunt the TPG, especially when aortic valve stenosis becomes critical.

**Figure 6:**
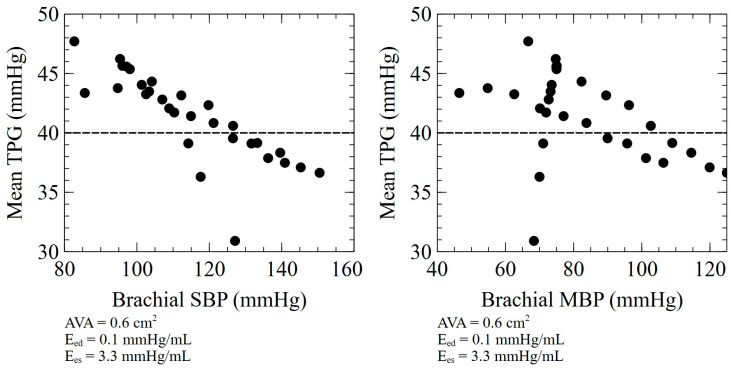
Association between peripheral blood pressure and mean TPG for cases presenting different combinations of TAC and TVR. SBP: Systolic blood pressure, MBP: Mean blood pressure.

Our simulation results confirm the independent effect of both components of arterial afterload on the TPG. Both impaired TVR and TAC were associated with a lower TPG for a given aortic valve stenosis. This association is also highly suggested by clinical and experimental data since the presence of arterial hypertension is associated with underestimated TPGs in patients with severe aortic stenosis [43,44]. In a recent study, the early return of arterial wave reflections (a parameter determined by both TAC and TVR) was associated with a lower TPG with a comparable AVA [45]. For these reasons, it is common in clinical practice to evaluate aortic valve stenosis after nitrates administration when the peripheral blood pressure is high. It is interesting to note, though, that the magnitude of the TPG effect from low TAC and high TVR was not comparable, with low compliance impacting the TPG more significantly than high resistance. Furthermore, different physiologic combinations of TAC and TVR may have different impacts on the TPG, even with comparable peripheral pressure values. The principle is shown in Figure 6, where cases with the same E_es_ and E_ed_ but different TAC and TVR combinations are plotted against peripheral blood pressures. It follows that the afterload evaluation (and thus the potential effect on the TPG) cannot be accurately predicted by measuring only peripheral blood pressures. For this reason, a comprehensive evaluation of left ventricular afterload could be particularly relevant when measuring the TPG. This can easily be achieved non-invasively through wave separation analysis by combining the pressure data obtained from a high-fidelity tonometer (the carotid or radial artery) and the aortic flow obtained by transthoracic echocardiography.

It is also interesting to note that the impact of E_es_, E_ed_, TAC, and TVR on TPG presents a significant interaction with the actual level of aortic stenosis since the effects seem to be more important as the aortic stenosis becomes more severe. This is of particular clinical importance, suggesting a greater risk for TPG underestimation in patients presenting with lower AVAs. At the same time, it is exactly these patients who require the most precise evaluation since the indication for valve replacement (vs. clinical follow-up) depends heavily on pressure gradients. Our proposed methodology offers a promising tool for distinguishing pseudo-stenosis from true stenotic cases. Future clinical studies are needed to validate our proposed methodology under different physiological conditions, such as decreased myocardial contractility or increased afterload. Additionally, the use of the model can be extended to other valve diseases as well.

## 5. Limitations

The conclusions of this current study are the results of mathematical simulation; thus, they should be interpreted with caution in the clinical setting. Isolated changes in E_es_, E_ed_, TAC, and TVR, although allowing for the precise estimation of the independent effect of each variable on pressure gradients, do not represent pure physiological states since significant interactions take place normally. Finally, the mathematical model does not incorporate physiological reflexes in response to blood and flow changes that may impact the TPG.

## 6. Conclusions

Both the systolic and diastolic ventricular functions have a profound effect on the TPG for a given aortic valve stenosis, leading to a potential underestimation of stenosis severity and a potential delay in therapeutic intervention. In the same direction, both TAC and TVR may blunt the TPG to the extent that it cannot be predicted solely based on the peripheral blood pressure levels. Moreover, the interdependence of the TPG’s left ventricular and afterload indices seem to intensify as aortic valve stenosis becomes more severe. A comprehensive evaluation of left ventricular function and afterload should be performed whenever possible, especially in cases of diagnostic challenges, such as paradoxical and classical low-flow, low-gradient stenosis, since it may offer the pathophysiological mechanism that explains the mismatch between aortic severity and the TPG. If it can be validated in future clinical trials, the proposed methodology will be a promising tool for differentiating patients with pseudo-stenosis from those with true stenosis.

## Figures and Tables

**Table 1:** Relative contribution of each determinant on TPG for different levels of aortic valve stenosis. Beta coefficients (Beta) are expressed in pressure (mmHg) for every 10% increase of each determinant from the baseline (lowest) value.

Aortic Valve Area	0.6 cm^2^			1.0 cm^2^			1.5 cm^2^			2.0 cm^2^		
	Beta	S.E.	*p* Value	Beta	S.E.	*p* Value	Beta	S.E.	*p* Value	Beta	S.E.	*p* Value
E_es_ (mmHg/mL)	3.4	0.1	<0.001	1.4	0.1	<0.001	0.7	0.1	<0.001	0.3	0.1	0.005
E_ed_ (mmHg/mL)	−5.6	0.5	<0.001	−2.8	0.2	<0.001	−1.3	0.1	<0.001	−0.6	0.1	<0.001
TAC (mL/mmHg)	1.3	0.2	<0.001	0.7	0.1	<0.001	0.6	0.1	<0.001	0.2	0.1	0.05
TVR (mmHg×s/mL)	−0.7	0.04	<0.001	−0.3	0.04	<0.001	−0.1	0.03	0.032	−0.04	0.04	0.321
Adjusted TVR * (mmHg×s/mL)	−1.1	0.04	<0.001	−0.8	0.1	<0.001	−0.4	0.03	<0.001	−0.2	0.02	<0.001

* coupled TVR according to its hyperbolic relation with TAC [61].

## Data Availability

The data presented in this study are available on request from the corresponding author.

## References

[1] Yu J., Wang Z., Bao Q., Lei S., You Y., Yin Z., Xie X. (2022). Global Burden of Calcific Aortic Valve Disease and Attributable Risk Factors from 1990 to 2019. Front. Cardiovasc. Med..

[2] Yadgir S., Johnson C.O., Aboyans V., Adebayo O.M., Adedoyin R.A., Afarideh M., Alahdab F., Alashi A., Alipour V., Arabloo J. (2020). Global, Regional, and National Burden of Calcific Aortic Valve and Degenerative Mitral Valve Diseases, 1990–2017. Circulation.

[3] Go A.S., Mozaffarian D., Roger V.L., Benjamin E.J., Berry J.D., Borden W.B., Bravata D.M., Dai S., Ford E.S., Fox C.S. (2013). Executive Summary: Heart Disease and Stroke Statistics—2013 Update: A Report From the American Heart Association. Circulation.

[4] Lindman B.R., Clavel M.-A., Mathieu P., Iung B., Lancellotti P., Otto C.M., Pibarot P. (2016). Calcific Aortic Stenosis. Nat. Rev. Dis. Primers.

[5] Roth G.A., Mensah G.A., Johnson C.O., Addolorato G., Ammirati E., Baddour L.M., Barengo N.C., Beaton A.Z., Benjamin E.J., Benziger C.P. (2020). Global Burden of Cardiovascular Diseases and Risk Factors 1990–2019. J. Am. Coll. Cardiol..

[6] Pibarot P., Clavel M.-A. (2022). Live Longer and Better without Aortic Valve Stenosis. Lancet Healthy Longev..

[7] Vahanian A., Beyersdorf F., Praz F., Milojevic M., Baldus S., Bauersachs J., Capodanno D., Conradi L., De Bonis M., De Paulis R. (2022). 2021 ESC/EACTS Guidelines for the Management of Valvular Heart Disease. Eur. Heart J..

[8] Baumgartner H., Hung J., Bermejo J., Chambers J.B., Edvardsen T., Goldstein S., Lancellotti P., LeFevre M., Miller F., Otto C.M. (2017). Recommendations on the Echocardiographic Assessment of Aortic Valve Stenosis: A Focused Update from the European Association of Cardiovascular Imaging and the American Society of Echocardiography. J. Am. Soc. Echocardiogr..

[9] Otto C.M., Nishimura R.A., Bonow R.O., Carabello B.A., Erwin J.P., Gentile F., Jneid H., Krieger E.V., Mack M., McLeod C. (2021). 2020 ACC/AHA Guideline for the Management of Patients With Valvular Heart Disease: A Report of the American College of Cardiology/American Heart Association Joint Committee on Clinical Practice Guidelines. Circulation.

[10] Reymond P., Bohraus Y., Perren F., Lazeyras F., Stergiopulos N. (2011). Validation of a Patient-Specific One-Dimensional Model of the Systemic Arterial Tree. Am. J. Physiol.-Heart Circ. Physiol..

[11] Kondiboyina A., Harrington H.A., Smolich J.J., Cheung M.M.H., Mynard J.P. (2022). Optimized Design of an Arterial Network Model Reproduces Characteristic Central and Peripheral Haemodynamic Waveform Features of Young Adults. J. Physiol..

[12] Charlton P.H., Mariscal Harana J., Vennin S., Li Y., Chowienczyk P., Alastruey J. (2019). Modeling Arterial Pulse Waves in Healthy Aging: A Database for in Silico Evaluation of Hemodynamics and Pulse Wave Indexes. Am. J. Physiol-Heart Circ. Physiol..

[13] Blanco P.J., Watanabe S.M., Passos M.A.R.F., Lemos P.A., Feijoo R.A. (2015). An Anatomically Detailed Arterial Network Model for One-Dimensional Computational Hemodynamics. IEEE Trans. Biomed. Eng..

[14] Bikia V., Adamopoulos D., Pagoulatou S., Rovas G., Stergiopulos N. (2021). AI-Based Estimation of End-Systolic Elastance from Arm-Pressure and Systolic Time Intervals. Front. Artif. Intell..

[15] Pagoulatou S., Rommel K.-P., Kresoja K.-P., von Roeder M., Lurz P., Thiele H., Bikia V., Rovas G., Adamopoulos D., Stergiopulos N. (2021). In Vivo Application and Validation of a Novel Noninvasive Method to Estimate the End-Systolic Elastance. Am. J. Physiol. Heart Circ. Physiol..

[16] Bikia V., Pagoulatou S., Trachet B., Soulis D., Protogerou A.D., Papaioannou T.G., Stergiopulos N. (2020). Noninvasive Cardiac Output and Central Systolic Pressure From Cuff-Pressure and Pulse Wave Velocity. IEEE J. Biomed. Health Inform..

[17] Clark C. (1978). Relation between Pressure Difference across the Aortic Valve and Left Ventricular Outflow. Cardiovasc. Res..

[18] Garcia D., Pibarot P., Durand L.-G. (2005). Analytical Modeling of the Instantaneous Pressure Gradient across the Aortic Valve. J. Biomech..

[19] Garcia D., Kadem L., Savéry D., Pibarot P., Durand L.-G. (2006). Analytical Modeling of the Instantaneous Maximal Transvalvular Pressure Gradient in Aortic Stenosis. J. Biomech..

[20] Hatoum H., Mo X.-M., Crestanello J.A., Dasi L.P. (2019). Modeling of the Instantaneous Transvalvular Pressure Gradient in Aortic Stenosis. Ann. Biomed. Eng..

[21] Ringle Griguer A., Tribouilloy C., Truffier A., Castel A.-L., Delelis F., Levy F., Vincentelli A., Bohbot Y., Maréchaux S. (2018). Clinical Significance of Ejection Dynamics Parameters in Patients with Aortic Stenosis: An Outcome Study. J. Am. Soc. Echocardiogr..

[22] Kim S.H., Kim J.S., Kim B.S., Choi J., Lee S.-C., Oh J.K., Park S.W. (2014). Time to Peak Velocity of Aortic Flow Is Useful in Predicting Severe Aortic Stenosis. Int. J. Cardiol..

[23] Altes A., Thellier N., Bohbot Y., Ringle Griguer A., Verdun S., Levy F., Castel A.L., Delelis F., Mailliet A., Tribouilloy C. (2021). Relationship Between the Ratio of Acceleration Time/Ejection Time and Mortality in Patients With High-Gradient Severe Aortic Stenosis. JAHA.

[24] Virag Z., Lulić F. (2008). Modeling of Aortic Valve Dynamics in a Lumped Parameter Model of Left Ventricular-Arterial Coupling. Ann. Univ. Ferrara.

[25] Aboelkassem Y., Savic D., Campbell S.G. (2015). Mathematical Modeling of Aortic Valve Dynamics during Systole. J. Theor. Biol..

[26] Laubscher R., van der Merwe J., Liebenberg J., Herbst P. (2022). Dynamic Simulation of Aortic Valve Stenosis Using a Lumped Parameter Cardiovascular System Model with Flow Regime Dependent Valve Pressure Loss Characteristics. Med. Eng. Phys..

[27] Korakianitis T., Shi Y. (2006). A Concentrated Parameter Model for the Human Cardiovascular System Including Heart Valve Dynamics and Atrioventricular Interaction. Med. Eng. Phys..

[28] Korakianitis T., Shi Y. (2006). Numerical Simulation of Cardiovascular Dynamics with Healthy and Diseased Heart Valves. J. Biomech..

[29] Mynard J.P., Davidson M.R., Penny D.J., Smolich J.J. (2012). A Simple, Versatile Valve Model for Use in Lumped Parameter and One-Dimensional Cardiovascular Models. Int. J. Numer. Methods Biomed. Eng..

[30] Mynard J.P., Smolich J.J. (2015). One-Dimensional Haemodynamic Modeling and Wave Dynamics in the Entire Adult Circulation. Ann. Biomed. Eng..

[31] Briand M., Dumesnil J.G., Kadem L., Tongue A.G., Rieu R., Garcia D., Pibarot P. (2005). Reduced Systemic Arterial Compliance Impacts Significantly on Left Ventricular Afterload and Function in Aortic Stenosis. J. Am. Coll. Cardiol..

[32] Hachicha Z., Dumesnil J.G., Pibarot P. (2009). Usefulness of the Valvuloarterial Impedance to Predict Adverse Outcome in Asymptomatic Aortic Stenosis. J. Am. Coll. Cardiol..

[33] Côté N., Simard L., Zenses A., Tastet L., Shen M., Clisson M., Clavel M. (2017). Impact of Vascular Hemodynamics on Aortic Stenosis Evaluation: New Insights Into the Pathophysiology of Normal Flow—Small Aortic Valve Area—Low Gradient Pattern. JAHA.

[34] Gardikioti V., Terentes-Printzios D., Iliopoulos D., Aznaouridis K., Sigala E., Tsioufis K., Vlachopoulos C. (2021). Arterial Biomarkers in the Evaluation, Management and Prognosis of Aortic Stenosis. Atherosclerosis.

[35] Gholampour S., Yamini B., Droessler J., Frim D. (2022). A New Definition for Intracranial Compliance to Evaluate Adult Hydrocephalus After Shunting. Front. Bioeng. Biotechnol..

[36] Gholampour S., Frim D., Yamini B. (2022). Long-Term Recovery Behavior of Brain Tissue in Hydrocephalus Patients after Shunting. Commun. Biol..

[37] Lancellotti P., Donal E., Magne J., Moonen M., O’Connor K., Daubert J.-C., Pierard L.A. (2010). Risk Stratification in Asymptomatic Moderate to Severe Aortic Stenosis: The Importance of the Valvular, Arterial and Ventricular Interplay. Heart.

[38] Mancusi C., de Simone G., Brguljan Hitij J., Sudano I., Mahfoud F., Parati G., Kahan T., Barbato E., Pierard L.A., Garbi M. (2021). Management of Patients with Combined Arterial Hypertension and Aortic Valve Stenosis: A Consensus Document from the Council on Hypertension and Council on Valvular Heart Disease of the European Society of Cardiology, the European Association of Cardiovascular Imaging (EACVI), and the European Association of Percutaneous Cardiovascular Interventions (EAPCI). Eur. Heart J.—Cardiovasc. Pharmacother..

[39] Gotzmann M., Hauptmann S., Hogeweg M., Choudhury D.S., Schiedat F., Dietrich J.W., Westhoff T.H., Bergbauer M., Mügge A. (2019). Hemodynamics of Paradoxical Severe Aortic Stenosis: Insight from a Pressure–Volume Loop Analysis. Clin. Res. Cardiol..

[40] Awtry E.H., Davidoff R. (2012). Low-Flow Low-Gradient Aortic Stenosis. Circ. Cardiovasc. Imaging.

[41] Burwash I.G., Pearlman A.S., Kraft C.D., Miyake-Hull C., Healy N.L., Otto C.M. (1994). Flow Dependence of Measures of Aortic Stenosis Severity during Exercise. J. Am. Coll. Cardiol..

[42] Burwash I.G., Thomas D.D., Sadahiro M., Pearlman A.S., Verrier E.D., Thomas R., Kraft C.D., Otto C.M. (1994). Dependence of Gorlin Formula and Continuity Equation Valve Areas on Transvalvular Volume Flow Rate in Valvular Aortic Stenosis. Circulation.

[43] Hayek A., Derimay F., Green L., Rosset M., Thibault H., Rioufol G., Finet G. (2020). Impact of Arterial Blood Pressure on Ultrasound Hemodynamic Assessment of Aortic Valve Stenosis Severity. J. Am. Soc. Echocardiogr..

[44] Kadem L., Dumesnil J.G., Rieu R., Durand L.-G., Garcia D., Pibarot P. (2005). Impact of Systemic Hypertension on the Assessment of Aortic Stenosis. Heart.

[45] Pagoulatou S., Adamopoulos D., Rovas G., Bikia V., Müller H., Giannakopoulos G., Mauler-Wittwer S., Licker M.-J., Stergiopulos N., Noble S. (2022). Arterial Wave Reflection and Aortic Valve Stenosis: Diagnostic Challenges and Prognostic Significance. Front. Cardiovasc. Med..

[46] deFilippi C.R., Willett D.L., Brickner M.E., Appleton C.P., Yancy C.W., Eichhorn E.J., Grayburn P.A. (1995). Usefulness of Dobutamine Echocardiography in Distinguishing Severe from Nonsevere Valvular Aortic Stenosis in Patients with Depressed Left Ventricular Function and Low Transvalvular Gradients. Am. J. Cardiol..

[47] Eleid M.F., Nishimura R.A., Sorajja P., Borlaug B.A. (2013). Systemic Hypertension in Low-Gradient Severe Aortic Stenosis With Preserved Ejection Fraction. Circulation.

[48] Reymond P., Merenda F., Perren F., Rüfenacht D., Stergiopulos N. (2009). Validation of a One-Dimensional Model of the Systemic Arterial Tree. Am. J. Physiol. Heart Circ. Physiol..

[49] Langewouters G.J., Wesseling K.H., Goedhard W.J. (1984). The Static Elastic Properties of 45 Human Thoracic and 20 Abdominal Aortas in Vitro and the Parameters of a New Model. J. Biomech..

[50] Holenstein R., Niederer P., Anliker M. (1980). A Viscoelastic Model for Use in Predicting Arterial Pulse Waves. J. Biomech. Eng..

[51] Womersley J.R. (1957). An Elastic Tube Theory of Pulse Transmission and Oscillatory Flow in Mammalian Arteries.

[52] Sagawa K., Maughan L., Suga H., Sunagawa K. (1988). Cardiac Contraction and the Pressure-Volume Relationship.

[53] Senzaki H., Chen C.-H., Kass D.A. (1996). Single-Beat Estimation of End-Systolic Pressure-Volume Relation in Humans. Circulation.

[54] Segers P., Stergiopulos N., Schreuder J.J., Westerhof B.E., Westerhof N. (2000). Left Ventricular Wall Stress Normalization in Chronic Pressure-Overloaded Heart: A Mathematical Model Study. Am. J. Physiol-Heart Circ. Physiol..

[55] Young D.F., Tsai F.Y. (1973). Flow Characteristics in Models of Arterial Stenoses—I. Steady Flow. J. Biomech..

[56] Young D.F., Tsai F.Y. (1973). Flow Characteristics in Models of Arterial Stenoses—II. Unsteady Flow. J. Biomech..

[57] Dekker A.L.A.J. (2003). Pressure-Volume Loops in Cardiac Surgery.

[58] Feldman M.D., Pak P.H., Wu C.C., Haber H.L., Heesch C.M., Bergin J.D., Powers E.R., Cowart T.D., Johnson W., Feldman A.M. (1996). Acute Cardiovascular Effects of OPC-18790 in Patients with Congestive Heart Failure. Time- and Dose-Dependence Analysis Based on Pressure-Volume Relations. Circulation.

[59] Chen C.H., Nakayama M., Nevo E., Fetics B.J., Maughan W.L., Kass D.A. (1998). Coupled Systolic-Ventricular and Vascular Stiffening with Age: Implications for Pressure Regulation and Cardiac Reserve in the Elderly. J. Am. Coll. Cardiol..

[60] Segers P., Stergiopulos N., Westerhof N. (2002). Relation of Effective Arterial Elastance to Arterial System Properties. Am. J. Physiol. Heart Circ. Physiol..

[61] Wohlfahrt P., Redfield M.M., Melenovsky V., Lopez-Jimenez F., Rodeheffer R.J., Borlaug B.A. (2015). Impact of Chronic Changes in Arterial Compliance and Resistance on Left Ventricular Ageing in Humans. Eur. J. Heart Fail..

[62] Baumgartner H., Hung J., Bermejo J., Chambers J.B., Evangelista A., Griffin B.P., Iung B., Otto C.M., Pellikka P.A., Quiñones M. (2009). Echocardiographic Assessment of Valve Stenosis: EAE/ASE Recommendations for Clinical Practice. J. Am. Soc. Echocardiogr..

[63] Pibarot P., Dumesnil J.G. (2013). Paradoxical Low-Flow, Low-Gradient Aortic Stenosis: New Evidence, More Questions. Circulation.

[64] Osnabrugge R.L.J., Mylotte D., Head S.J., Van Mieghem N.M., Nkomo V.T., LeReun C.M., Bogers A.J.J.C., Piazza N., Kappetein A.P. (2013). Aortic Stenosis in the Elderly: Disease Prevalence and Number of Candidates for Transcatheter Aortic Valve Replacement: A Meta-Analysis and Modeling Study. J. Am. Coll. Cardiol..

[65] Salmasi A.-M., Alimo A., Jepson E., Dancy M. (2003). Age-Associated Changes in Left Ventricular Diastolic Function Are Related to Increasing Left Ventricular Mass. Am. J. Hypertens..

